# Forces that shape the transcriptome: Linking cellular mechanosensing to mRNA splicing

**DOI:** 10.1016/j.jbc.2026.111352

**Published:** 2026-03-06

**Authors:** Pavel Simara, Cristina Mazzotti, Gokula Narayanan, Marco Cassani, Stefania Pagliari, Giancarlo Forte

**Affiliations:** 1 International Clinical Research Center (ICRC), St Anne’s University Hospital, Brno, Czech Republic; 2 Department of Biology, Faculty of Medicine, Masaryk University, Brno, Czech Republic; 3 School of Cardiovascular and Metabolic Medicine and Sciences, King’s College London, London, UK

**Keywords:** mechanosensing, RNA maturation, alternative splicing, RNA-binding proteins, mechanical stress

## Abstract

Mechanical signaling has been well documented in certain cell types, such as muscle cells, osteoblasts, fibroblasts, and other cells, historically defined as mechanocytes for their ability to receive and respond to mechanical stimuli. However, recent data suggest that mechanical signaling is not restricted to given cell types, but it is rather a universal feature of most eukaryotic cells that, similarly to extracellular chemical signaling, controls basic metabolic and intracellular signaling processes. Several studies published in recent years provided evidence that mRNA maturation is altered in cells exposed to mechanical stress. These data indicate that the process might be closely related to the 3D spatial reorganization of RNA-binding proteins. With mounting evidence for the mechanical control of mRNA splicing, this review aims to provide an overview of the available literature and offer a comprehensive vision of this phenomenon that stands out as a fundamental process in cellular biology.

In recent years, the role of mechanical forces in cellular function has shifted from a peripheral concept to a widely recognized phenomenon in molecular cell biology that has proven its relevance to medicine (1, 2). Mechanobiology—the study of how physical forces and changes in structure can influence cells and tissues—has uncovered critical links between tissue stiffness, shear stress, substrate elasticity, and the regulation of gene expression (3, 4, 5). While the effects of mechanical cues on DNA transcription are well documented, with the mechanical contribution to various aspects of cell behavior, including cell proliferation, differentiation, and migration, being well established, the influence of mechanical forces on RNA maturation is only beginning to emerge.

RNA maturation describes the intracellular processes that turn a just transcribed immature precursor mRNA (pre-mRNA) into a fully translatable mature mRNA. These processes include 5′ capping, splicing, and 3′ polyadenylation. Mature mRNAs are then transported to the cytoplasm for translation.

So far, convincing evidence has been provided for a role of mechanical stress in splicing. Although this evidence indicates that RNA capping, polyadenylation, and transport might also be shaped by mechanical forces, we will focus on mechanosensitive alternative splicing (AS). The term mechanosensitive RNA splicing refers to a cellular process originally identified by our group in the failing human heart as the direct consequence of the aberrant mechanical stress associated with extracellular matrix (ECM) pathological remodeling (6). The ensuing mechanical stress caused by ECM remodeling was compellingly shown to affect the nuclear localization and thus the function of heterogeneous nuclear ribonucleoprotein C (hnRNPC) in the splicing of thousands of mRNA transcripts. The identification of this new layer of mechanosensing based on the mechanical control of RNA-binding proteins (RBPs) adds to a decade of intense investigations on the mechanical control of DNA transcription (7).

At present, only two review articles have been published on this topic. In 2013, Liu and Tang (8) summarized the up-to-date reports on the effect of mechanical stress on AS and discussed the molecular mechanisms involved. Only lately, though, the role of mechanical forces on AS has been discussed in a review written by Bais and Giudice (9) after a decade of intense investigations. With increasing evidence on the importance of mechanical cues on the regulation of RNA maturation, we believe this emerging field deserves the attention of the scientific community.

In this review, we describe how mechanical signaling can now be considered a universal feature of eukaryotic cells that participates in developmental and adult functions to control basic metabolic and intracellular signaling processes. We proceed in establishing that RNA AS is needed for rapid adaptation of the organism to ensuing conditions by generating diverse mRNA transcripts from the same gene. Next, we critically discuss the hypothesis that mechanical stress rewires mRNA processing in a way that is crucial for development and pathology and introduce the importance of intranuclear 3D spatial organization of nuclear bodies for RNA splicing regulation. Finally, we focus on the factors influenced by mechanical stress that result in AS alterations in specific diseases described in emerging studies (6, 10, 11, 12, 13, 14, 15, 16, 17, 18) (Fig. 1).

**Figure 1:**
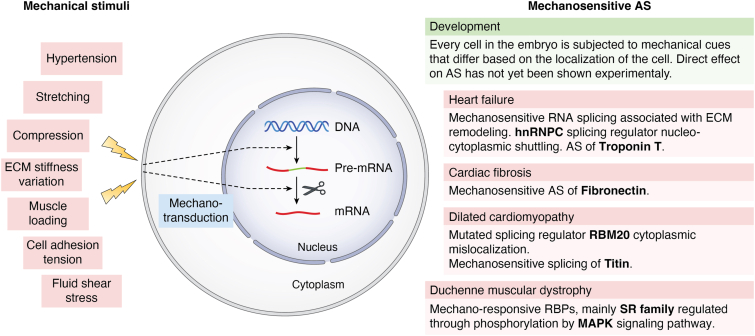
**The overview of mechanosensitive mRNA processing.** Schematic view of the effect of mechanical stimulation on mRNA processing in a way that is crucial for development (*green box*) and pathology (*red boxes*).

## Cellular mechanosensing

### Molecular mechanisms of cellular mechanosensing

In living cells, mechanical stimuli arise from the surrounding ECM or from neighboring cells. These stimuli may include mechanical stretching, ECM stiffness variation, cell-adhesion tension, pressure from neighboring cells, fluid shear stress, or others (Fig. 1). These forces act independently or in combination in a cell-specific context (19). The cell can sense mechanical cues mainly through dynamic integrin-based protein complexes called focal adhesions (FAs) (summarized by Martino *et al.* (4) and Oliver-De La Cruz *et al.* (3)) or cell–cell junctions (discussed in Ref. (20)).

**Figure 2:**
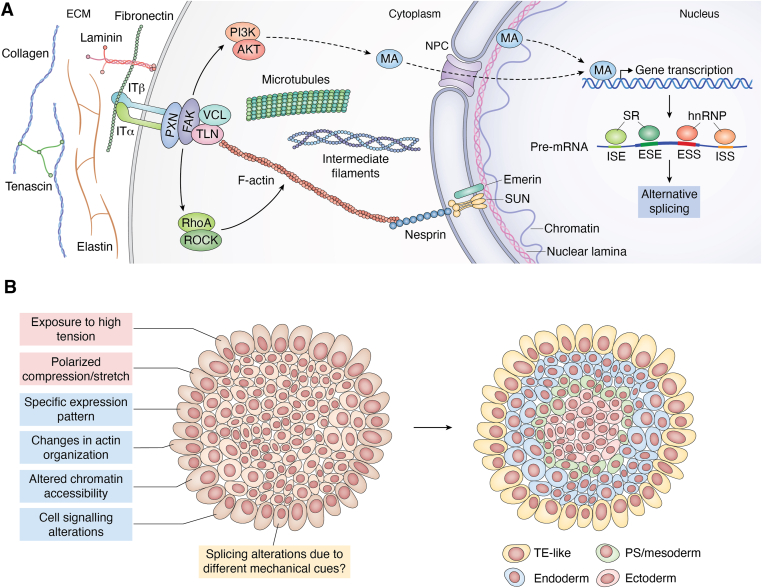
**Mechanical signaling and its effect on early differentiation.***A*, schematic representation of mechanosensing and mechanotransduction. The mechanical signals arising from the ECM are propagated by the cytoskeleton and are transferred into the nucleus, where genes are activated by MAs—shuttling mechanotransducers or mechanosensitive transcription factors. The splicing of pre-mRNA is regulated by RNA-binding proteins, mostly SRs and hnRNPs. Adapted from Oliver-De La Cruz *et al.* (3) and Martino *et al.* (4). *B*, the effect of spatial organization on the pluripotent stem cells (PSCs). The *cartoon* illustrates a 2D PSC colony cultured *in vitro*. The position of the PSCs within the colony and their exposure to a peculiar mechanical milieu affects their basic cellular processes (*left*) and primes them for differentiation (positional identity, *right*). Cell-adhesion tension and polarized compression/stretch are the main mechanical forces to which cells at the colony edge are exposed to. Colony-edge cells differ from colony-center cells in many aspects that eventually determine their fate, including mechanically induced AS, which was not yet proven experimentally. The radial differentiation model is adapted from the study by Warmflash *et al.* (65). AS, alternative splicing; ECM, extracellular matrix; ESE, exonic splicing enhancer; ESS, exonic splicing silencer; FAK, focal adhesion kinase; hnRNP, heterogeneous nuclear ribonucleoprotein; IF, intermediate filament; ISE, intronic splicing enhancer; ISS, intronic splicing silencer; IT, integrin; MA, mechanoactuator; MT, microtubule; NPC, nuclear pore complex; PS, primitive streak; PXN, paxillin; SR, serine/arginine-rich; TE, trophoectoderm; TLN, talin; VCL, vinculin.

ECM remodeling refers to changes in the biochemical and physical properties of the matrix, particularly the collagen network. Increased collagen deposition and crosslinking raise matrix stiffness, enhancing integrin-mediated force transmission and tensile stress experienced by cells compared with a soft matrix (21). Viscoelasticity is a feature of ECM and tissues that undergo time-dependent deformation upon external mechanical stimuli. Cellular tension is not sustained in soft viscoelastic matrices as the stress relaxation dissipates stress over time, whereas low viscous dissipation tends to maintain resistance and promotes prolonged force transmission (22). The directionality of collagen fiber arrangement (anisotropy) creates multiaxial stress around the cells and can alter the mechanical response. Alisafaei *et al.* (23) showed that tension anisotropy in fibroblasts is established by the interaction of cell protrusions with extracellular collagen fibers. This work shows that directional tension generated through ECM remodeling drives cell phenotypic transitions. Alterations in the ECM landscape can create topographical cues that influence key cellular events. Cells sense these topographical cues primarily through integrin-mediated interactions, which guide adhesion, migration, and cytoskeletal organization (24). We will focus here on ECM-generated forces perceived by FAs (Fig. 2*A*). FAs are divided into a transmembrane layer directly contacting the extracellular microenvironment and an intracellular layer connected to the actin cytoskeleton. On the molecular level, the mechanical cues are perceived by integrin clusters that interact with ECM proteins—mainly collagens, elastins, fibronectins, laminins, and tenascins (25). Integrin molecules transduce signals further to the intracellular FA core proteins that include focal adhesion kinase, talin, vinculin, and paxillin. The intracellular part of FA is connected to the cytoskeleton and serves as the second messenger of the mechanotransduction process. The cytoskeleton is a dynamic structure composed of actin fibers (F-actin), microtubules (MTs), and intermediate filaments. The transduction of the mechanical signal is ensured by structures called stress fibers, where F-actin slides on the motor protein myosin II. The last step is delivering the message to the nucleus by proteins shuttling through the nuclear envelope. A paradigm for such a mechanosensitive protein is the transcriptional coactivator Yes-associated protein (YAP), encoded by the *YAP1* gene (5, 26). YAP is a downstream effector of the Hippo pathway that is associated with cell proliferation and apoptosis inhibition in cancer, development, and organ size control (27, 28, 29). In addition to this, YAP directly promotes transcription of genes involved in cell–matrix and cell–cell interactions, ECM composition, cell spreading, and cytoskeleton integrity (5, 30, 31, 32, 33). Several other proteins were identified to shuttle into the nucleus upon mechanical stimuli, including ZO1, c-Abl, β-catenin, zyxin, and paxillin (summarized by Martino *et al.* (4)).

The nucleus itself displays a mechanosensitive apparatus of its own, named the nucleoskeleton. The force is transmitted to the nucleus by nuclear cytoskeletal coupling, where the nuclear envelope is a regulator of biological response (34). SUN and nesprin proteins are the main components of the so-called linker of the nucleoskeleton and cytoskeleton complex that forms a network of protein–protein interactions between the nuclear lamina and cytoskeleton (35, 36, 37). The main component of the nuclear lamina is protein lamin A that binds to chromatin either directly or through the regulatory protein emerin (38). Multiple studies have demonstrated the direct involvement of linkers of nucleoskeleton and cytoskeleton components and nuclear membrane proteins in the transcription regulation of genes associated with mechanosensing pathways (39, 40, 41, 42) or reduction of chromatin accessibility to transcription regulators (43). Experimental data indicate that cells regulate genomic programs *via* chromatin remodeling in response to mechanical changes of the surrounding microenvironment. Cell mechanical compression induces reversible chromatin condensation (44). On the other hand, in unconfined or stretched cells, rapid and transient chromatin decondensation was observed (45, 46). Expectedly, alterations in chromatin accessibility lead to gene transcription modulation. However, the different spatial distribution of active transcription sites within the nucleus implies changes in the localization of RNA-processing bodies, and, therefore, altered RNA splicing as a consequence of mechanical stimulation (47, 48).

While numerous transcriptional regulators have been associated with mechanical stimulation, insights into the mechanoregulation of splicing factors are still limited. The central pathway linking mechanical cues to AS in multiple cell types is the Rho–actomyosin signaling axis. Rho family GTPases respond to changes in ECM stiffness and adhesion tension by promoting actin polymerization and ROCK-dependent myosin light chain phosphorylation, which together drive actomyosin contractility and intracellular tension (49). Activation of RhoA enhances stress fiber assembly and contractile force generation, providing a mechanochemical platform that links extracellular forces to downstream biochemical signals (50). In osteoblastic cells, mechanical stimulation alters AS of vascular endothelial growth factor A in association with increased actin stress fiber formation, indicating that cytoskeletal tension can influence splice site selection in response to mechanical cues (15). In parallel, ECM stiffness regulates the mechanosensitive splicing factor PTBP1 through Rho-dependent actomyosin contractility. Increased intracellular tension affects PTBP1 nuclear localization and its control over AS of target transcripts, such as *Numb*, which is required for stiffness-induced cell spreading, proliferation, and osteogenic differentiation (18). Together, these findings indicate that actomyosin contractility downstream of Rho functions as a shared effector translating mechanical cues into AS programs across multiple cell types. In cancer cells, mechanical stiffening of the ECM also modulates AS through cytoskeletal tension and kinase signaling. Increased matrix stiffness enhances inclusion of the extra domain B (EDB) of fibronectin *via* serine/arginine-rich protein-mediated splicing, a process dependent on actomyosin contractility and PI3K–AKT activity. Cells cultured on stiff substrates exhibited higher levels of EDB-containing fibronectin compared with cells on compliant matrices, whereas reducing tumor stiffness *in vivo* decreases EDB inclusion, demonstrating that the biomechanical properties of the tumor microenvironment can bias splice site selection toward protumorigenic isoforms (51). In the failing heart, the PKC has been identified as an important mechanosignal transducer, leading to widespread changes in AS associated with cardiac dysfunction (6). Despite accumulating evidence that mechanical signals influence AS, the pathways and molecular principles by which these cues are translated into defined splicing programs remain fragmentary and incompletely characterized.

### Physiological mechanosensing

Biomechanical forces play a central role from the earliest stages of embryonic development, where tissues are constantly exposed to dynamic changes in fluid flow, extracellular osmolarity, and cycles of cellular stretching and contraction (52). One of the earliest morphogenetic events governed by mechanical cues is embryo compaction, during which extensive cell–cell contacts occur. Seminal work by Firmin *et al.* (53) demonstrated that cellular contractility in human embryos regulates the surface tension driving this compaction process and that defective contractility causes embryo loss. During the embryonic stage, originally homogenous pluripotent stem cells (PSCs (54, 55, 56)) are spatiotemporally differentiated to ensure proper tissue development. Recent studies highlighted the pivotal role of mechanical forces in regulating embryonic development. Already at the four-cell stage of *Caenorhabditis elegans* embryo development, AB and P lineage cells display distinct surface tensions that direct their fate (57). During gastrulation, mechanical stretching activates the β-catenin pathway to engage the Hippo pathway (see below) and control cytoskeleton dynamics. Eventually, this cascade leads to the promotion of mesoderm specification *via* the transcriptional activation of conserved target genes across species, such as *Drosophila*, *Zebrafish*, and *Nematostella* (58). In addition, the exclusive nuclear localization of the mechanically regulated YAP (see below) in the outside cells of the blastocyst suggests that mechanical forces might contribute to the specification of trophoectoderm and inner cell mass cells (59). Although previous studies indicate that splicing regulation during embryonic development might be substantially different from that occurring in the later stages of life (60), it is unclear whether mechanically induced RNA splicing is one of the molecular mechanisms that determine PSC heterogeneity. While the molecular determinants of PSC heterogeneity are mostly unknown, this phenomenon cannot be explained simply by differences in gene expression. Given the importance of RNA splicing in gene expression regulation, it is likely that AS is involved in mechanically induced control of cell pluripotency and differentiation.

Patterning that affects the cell potency and self-renewal was reported for *in vitro* cultured colonies of PSCs (61) (Fig. 2*B*). Kim *et al.* (62) showed that the cells at the edge of the colony display differential gene expression than the cells positioned in the colony center. The reason for this polarization is likely associated with mechanical forces. Treatment with tension inhibitor Y27632 (RhoA/ROCK inhibitor) led to perturbation in actin dynamics and disrupted the position-dependent polarity. In addition, the transcriptome analysis revealed genes that can be reliably used as markers of colony-edge cells (*TAGLN*) and colony-center cells (*NPTX1*), which have important practical implications for further research on cell spatial positioning. A similar observation was made about changes in actin organization resulting from distinct mechanical properties in the colony-edge PSCs, which were associated with early induction of differentiation at the colony perimeter (63). The mechanical heterogeneity within PSC colonies was confirmed by other studies, which demonstrated that the cells sitting on the colony border are exposed to higher tension compared with those located in the colony center (64). These studies indicate that cell fate specification is modified by mechanical forces that lead to changes in cell signaling, transcription, and tissue-specific differentiation. Wnt/β-catenin signaling was identified as the molecular determinant of this process, resulting in mesodermal differentiation. Other findings obtained by controlling PSC colony aspect ratio revealed that colony heterogeneity is reflected in the formation of concentric presumptive areas of specification, so that the same colonies display a spontaneous tendency to acquire a trophectoderm-like outer ring, an inner ectodermal circle, and a ring of mesendoderm-expressing primitive-streak markers in between (65). Interestingly, radially organized heterogeneity in PSC colonies persists even after cells detach and progress into 3D aggregates (66). During developmental transitions, mechanical forces integrate with morphogen signaling, as demonstrated by studies showing that bone morphogenetic protein 4 *per se* is not adequate to drive mesoderm induction and epiblast symmetry breaking in the absence of mechanical tension, highlighting the importance of mechanochemical cooperation in shaping early human development (67). The importance of mechanical forces is also increasingly recognized in other morphogenetic events, such as sprouting angiogenesis, the expansion of the vascular plexus from a pre-existing vessel network. Nishiyama’s laboratory recently showed that perivascular stiffening is a crucial factor regulating vascular branch elongation and lumen development. They also found that excessive lumen expansion is stymied by pericyte-driven endothelial deposition of collagen IV in the extracellular milieu (68). In addition to static and contractile forces, uniaxial cyclic stretching of epithelial cells has been shown to induce cell intercalation, which is a hallmark of morphogenesis (69). Another important implication of mechanical tension in the musculoskeletal developmental process was uncovered by Sunadome *et al.* (70), finding that, in zebrafish and mouse models, myofibril orientation is established through mechanically induced stretching and alignment of myoblast clusters.

The dominant role of mechanosensing is not limited to developmental aspects; it also plays a key role in immune response. Compelling research by Liu *et al.* (71) demonstrated that conventional antigen-presenting cells, particularly splenic dendritic cells 2, express the adhesion receptor CD97 (G-protein–coupled receptor family). Under blood flow–induced shear stress, circulating red blood cells expressing the ligand CD55 interact with CD97. This interaction exerts a pulling force on CD97, mechanically extracting its N-terminal fragment and thereby activating the receptor signaling cascade in dendritic cells. Disruption of this CD55–CD97 mechanosensory mechanism results in compromised lymphocyte responses to blood-borne antigens. The main immune cell patroller, the neutrophil, undergoes substantial deformation while traversing through narrow regions of pulmonary vessels. The mechanosensitive ion channel PIEZO1 has been shown to drive this neutrophil deformation–induced transcriptional reprogramming process in the pulmonary vasculature (72). Another study by Meizlish *et al.* (73) found that in 3D environments, macrophages sense the physical properties of their ECM and undergo cytoskeletal remodeling to drive the tissue repair process. Interestingly, this process occurs *via* “amoeboid migration,” which operates independently of integrin signaling.

Endothelial cells (ECs) lining the vasculature are constantly exposed to shear stress generated by blood flow. However, the molecular mechanisms underlying endothelial mechanosensing remain incompletely understood. Liu *et al.* (74) demonstrated that discoidin domain receptor 1 functions as a mechanosensor in ECs in response to flow. Under shear stress, discoidin domain receptor 1 forms condensates with the 14-3-3ε protein (YWHAE), which facilitates nuclear translocation of YAP (74). ECs respond to shear stress by inducing vasodilation to maintain vascular homeostasis. It has been discovered that plasma levels of EC-derived flow-sensitive epidermal growth factor–like domain–containing protein 1 (HEG1) were downregulated in patients with hypertension because of low wall shear stress (75). Collectively, these studies highlight mechanical forces as fundamental regulators of physiological function, shaping development, tissue morphogenesis, immune surveillance, and vascular homeostasis.

### Pathological mechanosensing

The ability of cells to respond to changes in their physical environment is critical for the development and maintenance of tissues exposed to variable mechanical stress. Therefore, any alteration in normal intracellular force transmission through changes in the intracellular or extracellular environment can lead to altered molecular forces, resulting in attenuation or enhancement of mechanosensitive mechanical signals. In addition to defects affecting cellular structure and organization and thus cellular mechanosensing, mutations in proteins involved in downstream signaling pathways could also cause impaired mechanotransduction. Alternatively, changes in the physical properties of the ECM could elicit pathological consequences, even when cellular mechanotransduction processes function properly, as excessive and continuous mechanical stimulation is associated with a wide range of diseases and pathological conditions. Common diseases like tumors, atherosclerosis, arthritis, osteoporosis, and cardiomyopathies, as well as various developmental disorders, like Hutchinson–Gilford progeria and Duchenne muscular dystrophy (DMD), involve abnormal physiological responses to mechanical forces (76, 77).

Comprising a complex meshwork of fibrillar and nonfibrillar collagens associated with growth factors and glycoproteins, the ECM plays a crucial role in maintaining the structural integrity and functionality of organs. When the tissue is injured, the repair process will be activated to rebuild the tissue structure by remodeling the ECM. Next, ECM will become stiffer because of changes in its composition and topology, and—in turn—these changes, together with the excessive ECM deposition, will eventually lead to fibrosis. Cardiac remodeling is a perfect model to study ECM composition and the impact of mechanics on organ physiology (78). Following myocardial infarction, for example, cardiac ECM undergoes significant changes in composition and structure because of cardiomyocyte loss. The scar tissue deposited by activated cardiac fibroblasts exhibits different mechanical properties, with increased stiffness, resulting in decreased heart tissue compliance (79). Changes in the ECM can also profoundly influence tumor progression. The tumor microenvironment is characterized by an altered ECM composition, which results in increased solid stress, promoting a more aggressive phenotype with increased metastatic ability and resistance to immune response and therapy (1). For instance, increasing ECM stiffness can activate YAP–transcriptional coactivator with PDZ-binding motif signaling, promoting cell proliferation and stemness of tumor cells (28, 80). ECM stiffening can also promote tumor progression *via* the activation of vinculin and PI3K, promoting basal membrane invasion (81). Moreover, ECM stiffness triggers Rho-dependent cytoskeletal tension, significantly reprogramming mitochondrial and metabolic states, meeting the high energy demands of metastasis and other energy-intensive activities (82). These alterations trigger rapid proliferation, enhanced invasion, and metastasis in various tumors (83, 84, 85).

The inflammatory effects of disturbed blood flow on the ECs are strikingly influenced by the underlying ECM. In unperturbed vessels, this matrix mainly consists of basement membrane proteins like collagen IV and laminins. Conversely, atherosclerosis-prone regions of arteries exhibit subendothelial fibronectin deposits, even in healthy mice (86). ECM remodeling together with shear stress triggers pathological effects by activating signaling pathways like NF-κB, transforming growth factor beta, YAP–transcriptional co-activator with PDZ-binding motif, PI3K–AKT, ALK5-Shc, and epigenetically inducing NF-κB and homeobox gene expression (87, 88). Recent studies have emphasized the role of classical mechanotransducers, including mechanosensitive channels, G-protein–coupled receptors, integrin adhesions, calcium channels, caveolae, the cytoskeleton, and the nucleoskeleton, along with their feedback mechanisms, in the pathological changes seen in atherosclerosis (89, 90).

Alterations in the mechanosensing process are involved in the development of neurodegenerative diseases. The cytoskeleton is essential for maintaining neuronal structure and function. In neurodegenerative diseases, like Alzheimer’s disease (AD) and Parkinson’s disease (PD), abnormal modifications of cytoskeletal proteins—Tau in AD or alpha-synuclein in PD—disrupt the normal dynamics and organization of MTs and actin filaments (91, 92). Furthermore, mutations in different genes and the alteration of proteins involved in mechanosensing and mechanotransduction lead to cytoskeleton instability, which contributes to the pathogenesis of the disease. For instance, mutations in Piezo1 may play a role in the development of AD, and the mechanosensitive ion channels TRPV4 and TREK-1, as well as integrins, have been suggested to be directly involved in the degeneration of retinal ganglion cells in glaucoma (93, 94).

## mRNA processing

mRNA processing is a fundamental step in gene expression, ensuring that primary transcripts are accurately and efficiently converted into mature, functional mRNAs. In eukaryotes, this involves several tightly regulated mechanisms, including 5′ capping, splicing, and 3′ polyadenylation (95, 96, 97). The 5′ cap protects mRNA from degradation and facilitates ribosome binding, whereas splicing removes introns and joins exons to generate coding sequences. AS further diversifies the transcriptome, allowing a single gene to produce multiple protein isoforms, some of which acquire distinct functions, a process that is particularly important during development. Polyadenylation at the 3′ end enhances mRNA stability and export from the nucleus. These processes involve dynamic interactions between RBPs and dedicated mRNA processing machineries, with splicing being regulated by the spliceosome and associated regulatory sequences. Disruptions in mRNA processing can lead to developmental defects and disease, underscoring its importance in embryonic and postnatal development (98). We will focus here on RNA splicing.

### Mechanism and regulation of RNA splicing

RNA splicing is a process that leads to the maturation of the pre-mRNA by removing introns (internal noncoding elements) and splicing back together exons (coding segments). The most common type of splicing in eukaryotes is carried out by the spliceosome, where two types are recognized: (i) constitutive splicing (CS) and (ii) AS. CS occurs typically when the sequence of the splice site is similar to the consensus sequence (strong splice site) and generates a single transcript. In AS, the splice sequence diverges from the consensus sequence (weak splice site), is suboptimally recognized by the spliceosome, and results in various isoforms of mature mRNA (99, 100). AS is used by the cells in all human organs to expand transcriptomic and proteomic diversity by synthesizing multiple protein variants from the same gene (96). According to genome-wide association studies, AS occurs in approximately 95% of all protein-coding genes in the human genome, although at different levels (101). AS allows cells to tailor protein function to meet the unique physiological demands of different tissues and developmental stages in higher eukaryotes.

The tightly orchestrated process of AS is catalyzed by the spliceosome, a dynamic macromolecular complex composed of various proteins and small nuclear RNAs (snRNAs), which assembles on the pre-mRNA molecule (102). The spliceosome assembly is governed by the *cis*-acting elements that are regulatory regions on pre-mRNA—exonic splicing enhancer or intronic splicing enhancer and exonic splicing silencer or intronic splicing silencer—together with the *trans*-acting factors that include proteins binding to the *cis*-acting elements (99, 100, 103, 104). The most extensively studied *trans*-acting factors are RBPs composed of the families of serine/arginine-rich (SR) proteins and hnRNPs. The SR proteins are best characterized for recognition and binding to splicing enhancers, whereas hnRNPs are the most prominent group of proteins recognizing splicing silencers. These two antagonistic families of RBPs are well conserved throughout eukaryotic evolution, and their interplay is believed to be a crucial factor in splicing regulation.

SR proteins share the ability to simultaneously bind RNA and other protein components, utilizing one or two N-terminal RNA recognition motifs (RRMs) and a C-terminal domain abundant in arginine and serine residues (RS domain), respectively. They participate in both CS and AS (105, 106) as well as mRNA nuclear export and translation (107). The main determinant of the SR protein activity is the location of its binding sites. In general, splicing is promoted when SR proteins bind to exons, whereas splicing is inhibited when SR proteins bind to introns (108). For the correct binding of SR proteins to the target RNA sequence, RS domain phosphorylation is required (109). Functional antagonists of SR proteins in splicing control are hnRNP proteins—large homopolymer complexes that reside predominantly in the nucleus. Besides their role in splicing, hnRNPs stabilize mRNA during their cellular transport and control their translation. In a steady state, hnRNPs are present in the nucleus; however, upon post-translational stimulation or by the recruitment of other hnRNPs, they can translocate to the cytosol (110). The members of the hnRNP protein family are named alphabetically from hnRNP A1 to hnRNP U, sharing a common structure and function. Only four unique RNA-binding domains (RBDs) were identified; therefore, multiple hnRNP proteins share the same RBDs. Their shared RNA-binding properties are RRM, quasi-RRM, and K-homology domain, RGG RBD consisting of Arg-Gly-Gly repeats (111). Many other RBPs are directly involved in the RNA splicing regulation process, for example, polypyrimidine tract–binding proteins (112), neuro-oncological ventral antigen proteins (113), and others (114).

### Organization of AS regulators in the nucleus

**Figure 3:**
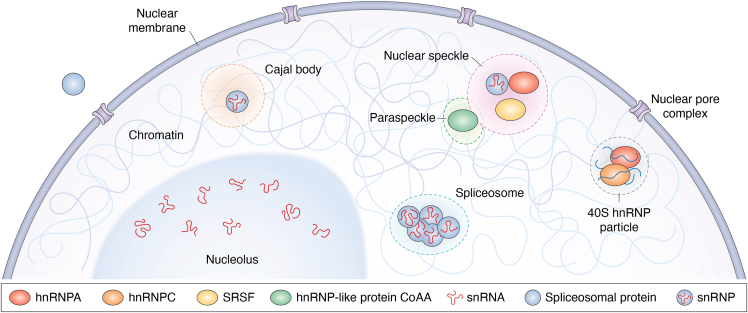
**Splicing components in the nucleus.** The *cartoon* shows part of the interphase nucleus with highlighted nuclear bodies that are involved in the process of RNA splicing control and selected splicing-regulatory proteins and snRNAs.

The regulatory components of the splicing machinery are well organized in various membraneless nuclear organelles that undergo dynamic exchange between each other and the nucleoplasm (115, 116) (Fig. 3). Among the most important bodies for splicing orchestration are nuclear speckles, also known as splicing speckles. Nuclear speckles are granule clusters in the interchromatin regions of the nucleus where pre-mRNA splicing factors are organized (115, 117). To analyze the spatial distribution of nuclear speckles, several components are commonly targeted by antibodies, for example, serine and arginine–rich splicing factor 1 or 2 (SRSF1 or SRSF2; SRSF2 is also known as SC-35), U2 small nuclear ribonucleoprotein (snRNP) component SF3a66, or AS regulator serine/arginine repetitive matrix 1 and 2 (47, 117, 118, 119). Both light and electron microscopy revealed that the speckled pattern is diffusely distributed throughout the nucleoplasm, often close to highly active transcription sites. However, previous studies show that transcription and splicing are not primarily located near speckles (119), which challenges the idea of nuclear speckles representing the sites of splicing. Nowadays, the nuclear speckles are thought to act as spliceosome storage assemblies and structural hubs that control the efficiency of RNA splicing. Bhat *et al.* (47) provided evidence that a gene transcribed from a genomic location proximal to nuclear speckles is more efficiently spliced than the same gene transcribed from a location farther from nuclear speckles. Changes in spatial gene organization relative to nuclear speckles subsequently lead to changes in splicing efficiency. Research by Paul *et al.* (118) demonstrated that the distinctive 3D organization of RNA molecules within the nuclear speckle is closely associated with differential proteome composition. Both SR and hnRNP families were associated with nuclear speckles—SRSF1 residing inside the speckle and hnRNPA1 outside the speckle. Their data suggest that RNA transcripts containing SR motif–enriched regions localize closer to the speckle center, whereas those enriched in hnRNP-binding motifs were found on the periphery of the speckle. Finally, evidence was provided to support a model where the intraspeckle RNA organization is determined by the interaction strength between RBP and RNA.

Besides the nuclear speckles, other well-described nuclear bodies have been associated with splicing—nucleoli, Cajal bodies, and paraspeckles (116). The nucleolus is the largest nuclear organelle and is primarily known as the site of ribosomal RNA synthesis and nascent ribosome assembly (120). The nucleolus is also involved in the trafficking of the spliceosomal U6 component, as U6 snRNA undergoes processing within this compartment (121). Cajal (coiled) bodies serve to assemble and transfer spliceosome components (snRNP particles) and modify snRNA. Mature snRNPs are transferred from the Cajal body into the nuclear speckles, whereas defective snRNPs are sequestered here (122). A key scaffolding protein that holds the whole particle together is coilin (123). Cajal bodies assemble at specific gene loci with high transcriptional activity, where they can shape the chromatin interaction landscape and the transcriptome by influencing spliceosome kinetics (124). Paraspeckles were—instead—discovered in 2002 and described as discrete bodies in the interchromatin nucleoplasmic space that are often located adjacent to nuclear speckles (125). Their main function is to control gene expression through polymerase II transcription and/or mRNA processing. A small number of proteins and RNAs are known to form paraspeckles, including transcriptional/splicing coregulator hnRNP-like protein CoAA (126, 127). Long noncoding RNA *NEAT1* serves—instead—as a scaffold for paraspeckle particles and represents a rare example of a scaffolding component based on a molecule of RNA (128).

Since some of the RBPs are known to form different functional complexes in the nucleus (129), less highlighted nuclear bodies exist and presumably more will be discovered in the future. For example, large complexes of hnRNP proteins assembled on pre-mRNA were discovered in 1977 by Beyer e*t al.* (130), termed 40S hnRNP particles. Only recently though, their complete protein composition has been determined (131), with hnRNPA and hnRNPC being identified as the major components, and RNA molecule as an essential scaffold that maintains the architecture of the 40S hnRNP particle. Altogether, these data indicate that splicing factors are dynamically organized within a 3D network of membraneless nuclear compartments, whose spatial architecture and RNA–protein interactions critically govern spliceosome availability, splicing efficiency, and transcript fate.

### AS in disease

AS is a key contributor to the diversity of the human proteome. The disruption of AS regulation can result in failing to meet cellular and tissue requirements, resulting in dysfunction and disease. Mutations affect splicing by disrupting either the *cis*-acting elements or the *trans*-acting factors, which leads to incorrect recognition or modulation of the splice site. Most disease-causing mutations in splicing proteins are autosomal dominant, with the disease arising from a mutation in only one copy of the gene (132, 133). The dominant nature of splicing factor mutations is expected, given the crucial role many of these proteins play in the splicing process. It is widely recognized that numerous human diseases stem from mutations that interfere with normal splicing patterns (134, 135). For example, mutations resulting in defective splicing constitute a significant proportion (48%) of a new series of mutations in the *ATM* gene in patients with ataxia–telangiectasia (136) and 50% of neurofibromatosis type 1 patients harbor mutations in the *NF1* gene that would lead to recurrent splicing alterations (137). Dysregulated AS is a common modulator or even a primary trigger in various tumors. Along with genetic mutations, epigenetic modifications, or dysregulated signaling pathways, malfunctioning AS may contribute to the well-known cancer-related mechanisms that include increased cell proliferation, decreased apoptosis, enhanced migration and metastasis, resistance to chemotherapy, and evasion of immune surveillance (138, 139, 140). An example is represented by the splicing of the p53 tumor suppressor.

**Figure 4:**
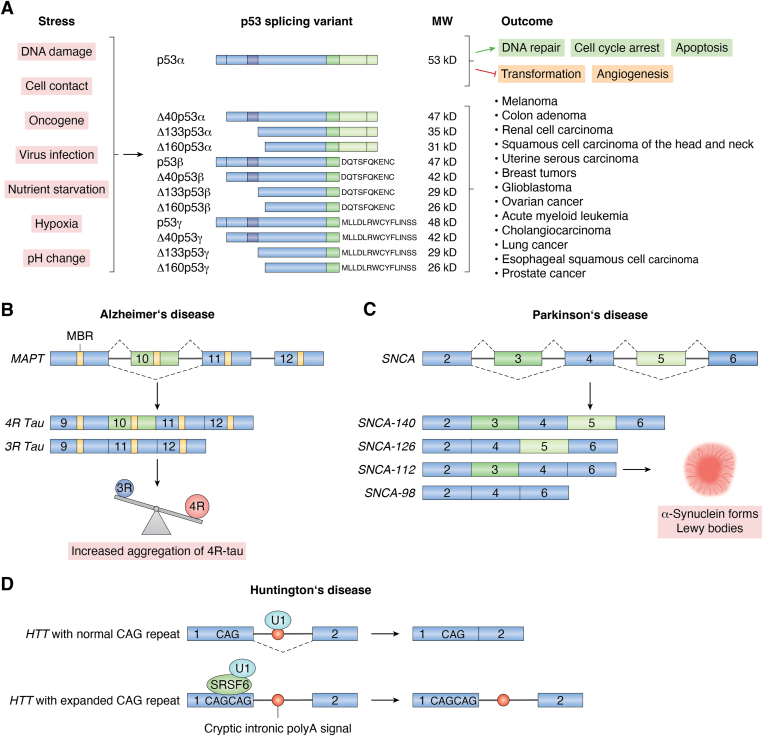
**Examples of disease-associated AS in cancer and neurodegenerative disorders**. *A*, p53 splicing. Twelve isoforms of p53 have been described so far, including the full-length wildtype p53α. Alternatively spliced isoforms have been associated with various human cancers. Adapted from Guo *et al.* and Surget *et al.* (141, 142). *B*, AS in Alzheimer’s disease 4R Tau protein isoform contains microtubule-binding regions encoded by four microtubule-binding repeats (MBRs), resulting from inclusion of alternative exon 10 in the *MAPT* gene. 4R Tau prevalence over 3R Tau variant results in aggregate formation and contributes to neurodegeneration. *C*, AS in Parkinson’s disease. Slice variants of the *SNCA* gene lead to accelerated formation of Lewy bodies through deposition of alpha-synuclein protein and promote the disease. *D*, AS in Huntington’s disease. *Lower*, SRSF6 splicing regulator recognizes the expanded CAG repeat in exon 1, which can interfere with U1 protection of polyA signals, leading to retention of intron 1. *Upper*, in a healthy *HTT* gene with a normal CAG repeat, U1 spliceosomal snRNP is protecting the cryptic intronic polyA signal, ensuring the properly spliced transcript. Adapted from Gipson *et al.* and Nikom and Zheng (143, 151). AS, alternative splicing; MBR, microtubule-binding repeat; snRNP, small nuclear ribonucleoprotein.

The p53 protein is encoded by the *TP53* gene and is often called the "guardian of the genome" because of its critical role in maintaining genomic stability in response to stress (Fig. 4*A*). The functional wildtype p53 (full length, also known as p53α) contains 11 canonical exons. Twelve distinct isoforms of p53 protein were described (141, 142). Alternatively spliced p53 isoforms can compete with the wildtype p53, which would effectively compromise the cell cycle control, apoptosis, and DNA repair and promote cell transformation and angiogenesis. Multiple studies link p53 isoform expression to clinicopathologic outcomes in human cancers (summarized by Guo *et al.* (141) and Fig. 4*A*).

The brain's high sensitivity to splicing errors is likely because of its complex variety of cell types and its dependence on splicing factors to adapt to the constantly changing molecular environment of the tissue. AS is crucial in many key neural developmental processes, and its dysfunction contributes to various neurodegenerative diseases, including AD, PD, Huntington’s disease, and others, where abnormal splicing patterns are frequently observed ((143); Fig. 4, *B*–*D*). What most alterations in splicing patterns have in common is the involvement of genes encoding for proteins with the tendency to create misfolded protein aggregates, hallmarks of various neurodegenerative pathologies. One of the clearest contributions of aberrant AS to AD-like tauopathy lies in the splicing of *MAPT*. The *MAPT* gene encodes the Tau protein, an MT-associated protein. Exon 10 of *MAPT* undergoes AS, producing Tau isoforms with either three (3R) or four (4R) MT-binding repeats (144). Normally, the ratio of 4R to 3R Tau is tightly regulated, but in AD, an imbalance favoring 4R Tau occurs, leading to increased aggregation and contributing to neurodegeneration (145, 146). Similarly, PD is strongly linked to the *SNCA* gene, which encodes alpha-synuclein, a protein involved in synaptic vesicle trafficking and neurotransmitter release. Certain splice variants may enhance the propensity of alpha-synuclein to aggregate in deposits called Lewy bodies, thereby accelerating their formation and exacerbating PD pathology (147). In addition, pathogenic expansions of simple sequence repeats, known as microsatellites, have been associated with over 30 neurological disorders (148, 149). Repeat expansions cause neurodegenerative diseases, mainly by forming toxic RNA foci that sequester splicing factors, hindering their ability to interact with their usual targets and disrupting normal splicing. In Huntington’s disease, an inherited expansion of CAG repeats in the *HTT* gene results in mutant *HTT* mRNA trapping spliceosomal proteins and AS regulators in a CAG repeat length–dependent manner, further contributing to splicing abnormalities and disease progression (150, 151).

### Mechanosensitive AS in the physiological state

In healthy organisms, mechanically induced AS is a crucial regulatory mechanism that contributes to tissue development, homeostasis, and physiological adaptation. This process is particularly vital in tissues constantly subjected to forces generated by fluid dynamics and tissue strain, allowing cells to translate mechanical information into functional protein diversity.

During embryo development at the cellular level, mechanical cues from the ECM are translated into cell fate decisions *via* the splicing machinery itself. The splicing factor PTBP1 acts as a key mechanotransducer, altering its nuclear abundance to control the splicing of the cell fate determinant Numb, thereby regulating proliferation and differentiation (18). During organogenesis, this mechanical instruction is fundamental for correct tissue architecture. In the developing lung, for instance, cyclic stretch from *fetal* breathing movements is required to direct the splicing of serum response factor, excising a "poison" exon to produce the full-length protein necessary for airway muscle formation (16). Similarly, the maturation of the inner ear, a process dependent on mechanical inputs, influences the AS of transmembrane channel-like 1, refining the biophysical properties of the auditory mechanotransduction channel (152).

Nowhere is the interplay between mechanics and developmental AS more evident than in the cardiovascular system, where the formation of blood vessels through vasculogenesis and angiogenesis is guided by mechanical forces. ECs sense shear stress generated by blood flow as well as the stiffness of the surrounding ECM, activating mechanotransduction pathways that modulate the activity of specific RBPs (153). These mechanically regulated splicing programs are essential for endothelial polarization, lumen formation, and vascular patterning. For example, the splicing regulator Nova2, whose expression and activity are controlled by mechanical cues in ECs. Nova2 directs the AS of multiple components of the Par polarity complex, including *Pard3* and *Pard6b*, a process that is required for EC polarization and the formation of a stable, hollow vascular lumen during development. Loss of Nova2-dependent splicing results in defective lumenization and aberrant vascular morphogenesis (154). In addition to regulating polarity, mechanosensitive AS can generate protein isoforms with distinct functional properties that refine the developing vascular network. For instance, Nova2-dependent splicing of the guidance receptor UNC5B produces a ligand-insensitive isoform that promotes endothelial apoptosis, contributing to vessel pruning during vascular remodeling (155).

The sustained increase in hemodynamic load after birth drives an RNA-binding motif protein 20 (RBM20)–mediated splicing switch in the giant protein titin. Titin is the longest protein in the human body, with a length exceeding 1 μm (156) and functions as a molecular spring spanning half of the sarcomere to connect the M-line to the Z-line and providing elasticity and stability to both cardiac and skeletal muscle (157). The titin is encoded by the *TTN* gene that consists of 364 exons, and the ratio between AS isoforms sets the passive stiffness of the muscle (158). In cardiomyocytes, AS of titin can be regulated mechanistically by substrate stiffness, resulting in AS variants N2B (shorter and stiffer) and N2BA (longer and more elastic) (10, 159). In mammalian fetal hearts, the long N2BA isoform, also called fetal cardiac titin, is predominantly expressed. After birth, fetal N2BA is gradually replaced with stiffer N2B, which is associated with increased myocardial passive stiffness. This event is critical for adapting the heart’s passive mechanical properties to the high-pressure postnatal circulatory system.

Importantly, RBM20 does not function solely as a static determinant of titin isoform choice. Rather, increasing mechanical load during postnatal maturation activates a broader RBM20-dependent splicing program that extends beyond *TTN* to include genes central to excitation–contraction coupling and calcium handling, such as *CAMK2D*, *CACNA1C*, and *RYR2*. Through coordinated AS of sarcomeric and calcium-regulatory proteins, mechanical cues are translated into transcriptome remodeling that synchronizes sarcomere stiffening with the maturation of contractile and electrophysiological properties of cardiomyocytes (160).

Beyond this developmental process, mechanosensitive AS is fundamental to the homeostatic maintenance and plasticity of the adult heart. In the mature myocardium, RBM20-dependent titin splicing remains dynamically regulated in response to physiological mechanical loads, such as those experienced during exercise, enabling fine-tuning of myocardial stiffness (161). Together, these findings demonstrate that mechanoregulated AS is a continuous process, translating physical forces into functional protein diversity from the earliest stages of embryo development through adaptive maintenance in adult tissues.

### Mechanosensitive AS in disease

Recent evidence suggests that the mechanical turmoil associated with ECM pathological remodeling might contribute to RNA splicing. Unsurprisingly, the most convincing data supporting the hypothesis of mechanically controlled RNA splicing come from the muscle, which itself produces and is exposed to continuous changes in force. Accumulating evidence suggests that there is a link between muscular and cardiac diseases, splicing misregulation, and mechanical alterations of the muscle (6, 12, 17, 158, 162, 163). However, little is known about the cooperation between mechanical forces and AS. Individual or global transcripts discussed in this chapter that were shown to be alternatively spliced upon mechanical stimulation are summarized in Table 1.

**Table 1:** Summarizes the individual or global transcripts that were shown to be alternatively spliced upon mechanical stimulation

Gene symbol	Gene full name	Exon count (National Center for Biotechnology Information)	Number of splice variants (ENSEMBL)	Type of mechanical stimulation	Regulator	Cell type	Clinical manifestation	References
FN1	Fibronectin 1	47	27	Myocardial injury, hypertension	ASF/SF2, hnRNP A1	Cardiac fibroblasts	Cardiac fibrosis, wound healing	White 2008 (169), Liao 2002, Ma 2012 (11)
TTN	Titin	364	16	Substrate stiffness variation	Rbm20	Cardiomyocytes	Heart failure, dilated cardiomyopathy	Nagueh 2004 (158), LeWinter (10) 2014, Li 2013 (162)
TNNT3	Troponin T3, fast skeletal type	21	19	Muscle loading; stretching	Akt pathway	Myoblasts	Skeletal muscle function	Schilder 2011, 2012 (12, 163)
IGF1	Insulin-like growth factor 1	7	7	Stretching	ASF/SF2	Osteoblast	Tissue growth and development, cancerogenesis regulation	Kasprzak and Szaflarski (13), Tang 2006 (180), Yi 2019 (181)
CCND1	Cyclin D1	5	6	Stretching	SRSF1, BAF57/SMARCE1	Osteoblasts, keratinocytes	Aberrant cell proliferation	Feng 2021 (14)
VEGFA	Vascular endothelial growth factor A	9	26	Stretching	F-actin polymerization, PI3K pathway	Osteoblasts	Osteogenesis, fracture healing	Faure 2008 (15)
SRF	Serum response factor	8	1	Stretching	Unknown	Mesenchymal cells	Lung hypoplasia	Yang 2000 (16)
Global	Global	NA	NA	Stretching	Mitogen-activated protein kinase pathway, SRSF4	Myoblasts	Muscular diseases	Hinkle 2022 (17)
YAP1	Yes1-associated transcriptional regulator	11	11	Substrate stiffness variation	hnRNPC	Cardiomyocytes, fibroblasts	Heart failure	Martino 2022 (6)
NUMB	NUMB endocytic adaptor protein	14	155	Cell density, size, and ECM stiffness variation	PTBP1	Mesenchymal progenitors and epithelial cells	Epithelial cell proliferation; mesenchymal stem cell differentiation	Tseng 2025 (18)

#### Heart failure

**Figure 5:**
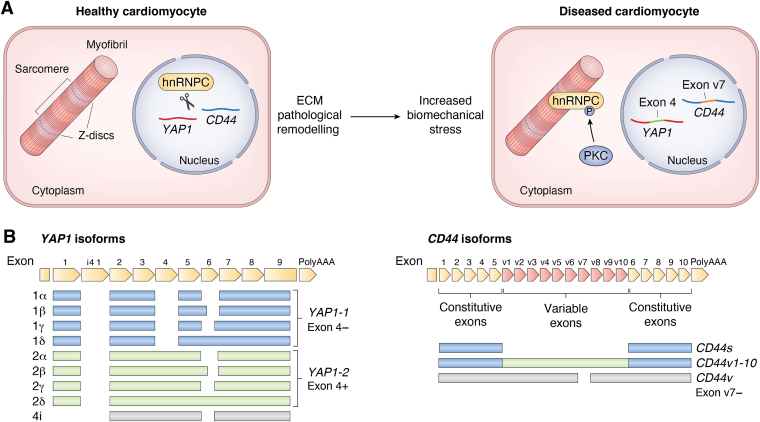
**Mechanosensitive AS in the failing heart**. *A*, mechanosensitive AS in the failing heart. Increased biomechanical stress generated by pathological cardiac ECM remodeling induces translocation of the splicing regulator hnRNPC from the cardiomyocyte‘s nucleus to the sarcomeric Z-discs—an active site of localized translation. The depletion of hnRNPC from the nucleus leads to substantial changes in RNA splicing, including the retention of exon 4 in *YAP1* and variable exon 7 in *CD44* transcripts. hnRNPC intracellular localization is mechanically controlled *via* PKC phosphorylation. Adapted from Martino *et al.* (6). *B*, AS of *YAP1* and *CD44*. Adapted from Vrbsky *et al.* and Schmitt *et al.* (165, 201). AS, alternative splicing; ECM, extracellular matrix; hnRNPC, heterogeneous nuclear ribonucleoprotein C.

While several studies found SR proteins to be responsible for AS induced by mechanical stimuli, there is less evidence for the involvement of their RBP counterparts—hnRNP proteins—in the mechanoregulated AS. Recently, our group used human heart tissue and cell cultures to describe the mechanosensitive RNA splicing associated with ECM remodeling (6). We showed that the shuttling of hnRNPC from the nucleus of cardiomyocytes is mechanically induced. hnRNPC translocates to sarcomeric Z-discs and associates with the translation machinery (Fig. 5*A*). The depletion of hnRNPC from the nucleus directly affects the ability of hnRNPC to regulate AS of pre-mRNAs. In fibroblast cells and cardiomyocytes derived from human-induced PSCs, knockdown and pharmacological inhibition of hnRNPC showed different splicing of genes involved in cell mechanotransduction, integrin-mediated cell adhesion, and FA. *YAP1* was also among the differentially spliced genes. Specifically, hnRNPC depletion promoted the inclusion of exon 4 in the *YAP1* transcript and affected the overall balance among *YAP1-1* and *YAP1-2* splicing variants (Fig. 5*B*). In genetically modified cell lines in which hnRNPC was targeted to the cytoplasm only through the introduction of a point mutation in the nuclear localization signal, the inclusion of exon 4 into the *YAP1* transcript was observed, proving that the mechanical displacement of hnRNPC from the nucleus affects the splicing of *YAP1*.

Similar results were obtained for CD44, a cell-surface glycoprotein involved in cell–cell interactions, cell adhesion, and migration, whose AS has been previously identified as mechanosensitive (164). *CD44* has a total of 20 exons and generates multiple isoforms through AS of 10 variable exons (v1–10), including the standard form (*CD44s*, lacking variable exons) and numerous variant isoforms (*CD44v*) (165) (Fig. 5*B*). The inclusion of variable exon 7 into the *CD44* transcript increased upon hnRNPC depletion and upon treatment with inhibitors of tension. The comparison of differentially spliced genes in hnRNPC-depleted cells to the RNAs known to harbor hnRNPC-binding sites (POSTAR3 database) revealed that 85% of the transcripts had at least one hnRNPC-binding site. Using individual-nucleotide resolution crosslinking and immunoprecipitation analysis, 748 transcripts were found to physically interact with hnRNPC in fibroblast cells that were differentially spliced in the absence of the protein. Overall, this study was the first to demonstrate how ECM remodeling triggers cardiac cell fast adaptation in response to mechanical stimuli by taking advantage of mechanosensitive RBP and AS in heart failure.

Another evidence for muscle gene transcripts undergoing AS upon mechanical stimuli comes from the studies on troponin T (TnT). TnT is the tropomyosin-binding subunit of the troponin complex and is integral to skeletal and cardiac muscles (166). Mammalian skeletal muscle TnT is encoded by a slow (*TNNT1*) and fast (*TNNT3*) gene, whereas cardiac troponin is encoded by the *TNNT2* gene (167). The research of Schilder *et al.* (12) demonstrated that the TnT3 subunit undergoes extensive AS in response to experimentally induced increases in muscle loading in rats or mechanical stretch of C2C12 mouse myoblasts (163). The major role in muscle plasticity and calcium sensitivity is adjusted by the inclusion of exon 4 after mechanical stimulation. Their data suggest that AS is controlled by a cell-autonomous mechanism through Akt signaling but not ERK1/2. *TNNT2* gene, encoding cardiac TNT (cTnT), undergoes AS primarily affecting exons 4 and 5, leading to the production of four human cardiac isoforms: cTnT1 through cTnT4 (166). The fetal heart predominantly expresses cTnT1 and, to a lesser extent, cTnT2 and cTnT4, whereas cTnT3 becomes the human adult heart–specific isoform. Interestingly, the cTnT4 isoform expressed in the fetal heart becomes re-expressed in failing human hearts (166).

#### Cardiac fibrosis

Cardiac fibroblasts are the main producers of ECM in the heart, responsible for tissue repair and remodeling mechanisms, especially after myocardial injury or in response to chronic mechanical stress, such as hypertension. Cardiac fibroblasts in the heart are highly sensitive to mechanical cues, and these cues significantly influence the splicing of fibronectin pre-mRNA. The *FN1* gene is composed of three types of modules: type I, type II, and type III repeats, encoding for functional domains arranged differently to give rise to soluble or insoluble fibronectin proteins. The inclusion or exclusion of these domains is controlled by AS, leading to different fibronectin isoforms. In response to increased mechanical load, such as during cardiac hypertrophy or following myocardial infarction, cardiac fibroblasts increase the production of specific fibronectin isoforms, including those with the extra domain A (EDA) of FN, which are associated with a fibrotic response (11). The inclusion of the EDA results in a more cell-adhesive form of fibronectin (168), enhancing the ability of fibroblasts to generate a more fibrotic and stiffer ECM, contributing to pathological cardiac remodeling, especially in the case of cardiac hypertrophy (11). The splicing of the EDA exon is positively regulated by the splicing factor ASF/SF2, and its skipping is under the control of hnRNPA1 (169). In addition to cardiac fibroblasts, the mechanosensitive splicing of fibronectin can also occur in other cell types, such as ECs and smooth muscle cells, under specific conditions, where these cells are exposed to mechanical forces (11). However, in the context of the heart, fibroblasts are credited with being the primary cell type involved in this process.

#### Dilated cardiomyopathy

The splicing repressor RBM20 is a member of the SR protein family (162) that is predominantly expressed in the heart, and its mutations were associated with human dilated cardiomyopathy (DCM) (170, 171, 172, 173). The majority of DCM-causing RBM20 variants are heterozygous missense mutations, which cluster in a conserved stretch encoding for six amino acids, PRSRSP, in the protein’s RS-rich domain (171, 174, 175, 176). Pathogenic variants in *RBM20* account for approximately 2% to 6% of the cases of familial DCM with early onset heart failure and high mortality (177). Loss-of-function mutation in the *RBM20* gene in both human and rat results in DCM and arrhythmia with sudden death (177). While the wildtype RBM20 protein localization is exclusively nuclear, mutations in *RBM20* often result in cytoplasmic mislocalization and granule formation, which leads to splicing defects (170). Interestingly, the splice-regulatory activity of RBM20 mutants is proportional to their nuclear localization (170). Although the disease-causing mutation is only present in cardiomyocytes, recent data show that it alters the functioning of surrounding fibroblasts, resulting in ECM stiffening (178). The most prominent splicing target of RBM20 associated with DCM is titin, whose role in physiological heart development has been discussed in the previous section. Remarkably, the diseased adult heart undergoes a reverse titin isoform transition. In this regard, in transplanted hearts of coronary artery disease patients, and in patients with DCM and heart failure, the fraction of the fetal titin isoform N2BA is substantially higher than in controls (157). An upregulation of the N2BA isoform is therefore compensatory, counteracting the increased stiffness of the ECM and seeming to act as an adaptive response to disease to improve cardiac function (157). Besides titin, RBM20 regulates splicing of the genes involved in mitochondrial function (*e.g.*, the *IMMT* gene encoding the mitochondrial inner membrane protein), calcium handling (*e.g.*, the *RYR2* gene encoding the ryanodine receptor 2 in cardiac muscle), and ion channels (*e.g.*, the *CACNA1C* gene encoding the subunit of the L-type voltage–dependent calcium channel) (161, 175, 179).

#### Duchenne muscular dystrophy

Mechanosensitive RBPs were recently described by Hinkle *et al.* (17). DMD is known to dysregulate the splicing pattern in mice with DMD, which also display alterations in mechanosensitive signaling pathways, as revealed by deep sequencing. Stretching C2C12 muscle cells for hours (1–6 h) leads to changes in the expression of components of the mitogen-activated protein kinase pathway, which in turn promotes the AS of cassette exons and favors exon inclusion over skipping, where RBPs play a role. SR proteins are known to respond to stretching more than other RBP families. For example, mechanical cues regulate SRSF4 phosphorylation, which interacts with members of the mitogen-activated protein kinase signaling pathway (17).

#### Mechanosensitive AS in osteoblasts and other cell types

Well documented is the effect of mechanical stress on the AS of insulin-like growth factor 1 (IGF1), which balances the expression of three splicing variants, IGF1Ea, IGF1Eb, and IGF1Ec (13). The splicing variant IGF1Ec, also known as mechanogrowth factor, was shown to be highly expressed in rat osteoblasts in response to mechanical stress—cyclic stretching (180, 181). Similarly to the data obtained on muscle cells, RBPs belonging to the SR protein family, ASF/SF2 in this case, were identified as the ones controlling the IGF1 AS induced by mechanical stimuli in osteoblasts.

Osteoblast and keratinocyte cells were instead exposed to cyclic stretch and produced evidence pointing to the existence of mechanically controlled cyclin D1 AS (14). The results indicated that the mechanical splicing of cyclin D1 was mediated by splicing factors SRSF1 and BAF57/SMARCE1. In other studies, mechanical stimulation by stretching was also associated with changes in AS variants of proteins like serum response factor (in smooth muscle cells), tension-induced/inhibited proteins (in lung embryonic mesenchymal cells), and vascular endothelial growth factor (in osteoblastic cells) (15, 16, 182). All the abovementioned studies concurred to identify splicing events in transcripts of individual genes induced by mechanical stimuli. Other studies focused more on global AS patterns.

Mechanosensitive behavior was recently reported for another member of the hnRNP protein family, PTBP1 (also known as hnRNPI), in mesenchymal progenitors and epithelial cells (18). Upon mechanical stimulation, such as changes in cell density, size, and ECM stiffness, PTBP1 changes its nuclear abundance in a similar manner as was described for hnRNPC in heart cells (6) and, in general, for the YAP transcriptional regulator (5, 26). The endocytic adapter Numb, a tumor suppressor whose splicing alterations have been associated with cancer-promoting activity, was identified as a major splicing target of PTBP1 (183). These data shed light on the involvement of mechanically regulated splicing in the osteoblast and adipocyte differentiation process as well as in cancer progression.

### The interconnection of mechanosensing and RBPs in the control of post-transcriptional events

Around 1000 RBPs have been identified to date as effectors along the entire gene expression pathway that includes AS, mRNA stability, and translation (184). Several recent publications highlight the ability of RBPs to respond to the mechanical stimulation that eventually results in changes in AS. The most prominent examples were discussed above—mechanosensitive phosphorylation of SRSF4 (17) or mechanically induced nucleocytoplasmic shuttling of hnRNPC (6) and PTBP1 (18). In these cases, the modulation of RBP behavior by mechanical cues has implications for diseases. Beyond AS, important molecular mechanisms mediating eukaryotic pre-mRNA processing are 5′ m7G capping and 3′ polyadenylation. The alternative polyadenylation (APA) provides mRNA stability and results in numerous transcripts with differing 3′ ends, thus greatly expanding the diversity of mRNAs (185). Multiple RBP-binding sites are located in the 3′UTRs of RNA, and the regulation of RBP–RNA binding is crucial in various diseases. For example, hnRNP A18 (also known as CIRP) targets 3′UTR motifs on the mRNA of cancer-associated prosurvival genes and stabilizes them (186, 187). Another highly conserved RBP, Musashi1 (Msi1), functions as a regulator of translation by binding to specific motifs located in the 3′UTRs of its target mRNAs (188, 189). Msi1 promotes tumorigenesis and is highly expressed across diverse tumor types. Interestingly, the 3′UTR sequence of *Msi1* mRNA itself can be targeted by another important RBP called HuR that is associated with glioblastoma (190). Changes in APA have also been found in cardiac hypertrophy (191) and heart failure (192). The roles of 5′ m7G capping are initiation of protein synthesis, protecting the mRNA molecule from exonuclease cleavage, and recruiting proteins for pre-mRNA splicing, polyadenylation, and nuclear export (reviewed in Ref. (193)). The cap structure is directly involved in the mRNA splicing and 3′-end formation processes through binding to the nuclear cap-binding complex (194, 195). Unlike for AS, there is no direct evidence linking mechanical signaling to mRNA stability or translation through APA or m7G capping to date. However, emerging studies reporting the mechanically induced response of RBPs together with their key role in controlling APA suggest that such a connection might be revealed soon.

### Indirect effects of mechanical forces on AS through physical changes of the nuclear shape

Mechanical cues can alter AS indirectly by influencing the spatial organization of the nucleus and chromatin condensation. Mechanical forces, such as those experienced during cell migration, tissue stretching, or fluid shear stress, can result in significant changes in nuclear shape (196). Changes in nuclear shape can lead to the repositioning of chromatin domains and nuclear bodies relative to the nuclear periphery (197). This repositioning can alter the accessibility of splicing regulatory elements and the efficiency of spliceosome assembly at specific gene loci, thereby influencing AS outcomes (196). The disruption in lamin A/C, a major component of the lamina, can change the association of chromatin with the lamina, thereby affecting the splicing of lamina-associated genes, particularly under mechanical stress (198). Direct structural changes in nuclear bodies involved in splicing control were reported after mechanical stress application by *Poh et al.* (199). In their experiment, a surface force/deformation *via* integrins altered the shape of the nucleus and resulted in displacements of coilin and SMN proteins in Cajal bodies. In addition, mechanical cues can change the accessibility of splicing regulatory elements, promoting chromatin remodeling (200). Chromatin exists in a dynamic equilibrium between condensed (heterochromatin) and relaxed (euchromatin) states, and this balance can be modulated by mechanical cues. For instance, stretching cells induces chromatin decondensation, which correlates with the activation of specific AS events (45, 46, 200).

## Conclusion and future perspectives

As discussed above, accumulating evidence suggests that mechanical signaling plays an important role in regulating RNA maturation. Although convincing evidence has been provided that mechanical stress rewires RNA splicing only, effects on the other phases of the maturation process are not to be excluded. AS dysregulation has been associated with various diseases, including muscular and cardiac diseases, neurodegenerative disorders, and a wide range of tumors. A better understanding of how mechanical stimuli influence AS can lead to the development of new treatment options. These might target members of the mechanotransduction pathways or splicing processes through small molecules, gene editing technologies, or antisense oligonucleotides.

Developmental biology is another field where studying the mechanical regulation of RNA splicing might bring novel insights. Mechanosensitive AS is likely to contribute to the positional identity of cells located in different areas of the PSC colony and thus to PSC heterogeneity. Studying this phenomenon could help to reveal the key biological mechanisms governing cell differentiation and enhance our fundamental knowledge of cell development.

In summary, mechanical stress affects RNA expression by altering AS. These alterations can have profound effects on gene expression, influencing how cells respond to their mechanical environment, particularly in mechanically active tissues, such as muscle, bone, and the cardiovascular system. Understanding these processes at a molecular level will provide deeper insights into how mechanical forces contribute to normal physiology and disease.

## Conflict of interest

The authors declare that they have no conflicts of interest with the contents of this article.
